# Effects of Monitoring Frailty Through a Mobile/Web-Based Application and a Sensor Kit to Prevent Functional Decline in Frail and Prefrail Older Adults: FACET (Frailty Care and Well Function) Pilot Randomized Controlled Trial

**DOI:** 10.2196/58312

**Published:** 2024-10-22

**Authors:** Myriam Valdés-Aragonés, Rodrigo Pérez-Rodríguez, José Antonio Carnicero, Pedro A Moreno-Sánchez, Myriam Oviedo-Briones, Elena Villalba-Mora, Pedro Abizanda-Soler, Leocadio Rodríguez-Mañas

**Affiliations:** 1 Geriatrics Service Getafe University Hospital Getafe Spain; 2 Intelligent Robotics Lab Universidad Rey Juan Carlos Fuenlabrada Spain; 3 Biomedical Research Foundation Getafe University Hospital Getafe Spain; 4 Faculty of Medicine and Health Technology Tampere University Tampere Finland; 5 Human-Computer Interaction Laboratory Centre for Biomedical Technology Universidad Politécnica de Madrid Madrid Spain; 6 Center of Biomedical Research on Bioengineering, Biomaterials and Nanomedicine Instituto de Salud Carlos III Madrid Spain; 7 Geriatrics Service Albacete University Hospital Albacete Spain; 8 Center of Biomedical Research on Frailty and Healthy Aging Instituto de Salud Carlos III Madrid Spain

**Keywords:** frailty, functional status, older adults, new technologies, sensor, monitoring system, information and communication technologies, mobile app, sensor kit, sensors, technological ecosystem, clinical intervention

## Abstract

**Background:**

Frailty represents a state of susceptibility to stressors and constitutes a dynamic process. Untreated, this state can progress to disability. Hence, timely detection of alterations in patients’ frailty status is imperative to institute prompt clinical interventions and impede frailty progression. With this aim, the FACET (Frailty Care and Well Function) technological ecosystem was developed to provide clinically gathered data from the home to a medical team for early intervention.

**Objective:**

The aim of this study was to assess whether the FACET technological ecosystem prevents frailty progression and improves frailty status, according to the frailty phenotype criteria and Frailty Trait Scale-5 items (FTS-5) at 3 and 6 months of follow-up.

**Methods:**

This randomized clinical trial involved 90 older adults aged ≥70 years meeting 2 or more Fried frailty phenotype criteria, having 4 or more comorbidities, and having supervision at home. This study was conducted between August 2018 and June 2019 at the geriatrics outpatient clinics in Getafe University Hospital and Albacete University Hospital. Participants were randomized into a control group receiving standard treatment and the intervention group receiving standard treatment along with the FACET home monitoring system. The system monitored functional tests at home (gait speed, chair stand test, frailty status, and weight). Outcomes were assessed using multivariate linear regression models for continuous response and multivariate logistic models for dichotomous response. *P* values less than .05 were considered statistically significant.

**Results:**

The mean age of the participants was 82.33 years, with 28% (25/90) being males. Participants allocated to the intervention group showed a 74% reduction in the risk of deterioration in the FTS-5 score (*P*=.04) and 92% lower likelihood of worsening by 1 point according to Fried frailty phenotype criteria compared to the control group (*P*=.02) at 6 months of follow-up. Frailty status, when assessed through FTS-5, improved in the intervention group at 3 months (*P*=.004) and 6 months (*P*=.047), while when the frailty phenotype criteria were used, benefits were shown at 3 months of follow-up (*P*=.03) but not at 6 months.

**Conclusions:**

The FACET technological ecosystem helps in the early identification of changes in the functional status of prefrail and frail older adults, facilitating prompt clinical interventions, thereby improving health outcomes in terms of frailty and functional status and potentially preventing disability and dependency.

**Trial Registration:**

ClinicalTrials.gov NCT03707145; https://clinicaltrials.gov/study/NCT03707145

## Introduction

Functional status is one of the best indicators of health condition and predicts poor outcomes better than morbidity in older people [1]. In the pathway leading from robustness to disability, frailty is a dynamic process, and transitions that occur in the state of frailty happen bidirectionally: from better to worse states on the spectrum of frailty as well as toward disability [2]. Life expectancy has increased in the recent decades [3], leading to an increase in the older population, which, in turn, has led to an increase in the number of frail people. Therefore, prevention and treatment of frailty have generated great interest since it represents a challenge for health systems [4]. Several studies have shown that multimodal interventions, that is, those targeting multiple factors are effective both in primary (prevent or delay the debut of frailty) and secondary prevention (treating the syndrome when it has appeared with the aim of reversing it as much as possible) of frailty, particularly in older individuals with multimorbidity and high health care utilization [5,6].

The current medical practice to assess, treat, and monitor frailty is performed through periodic visits of the patient to health care settings. Between these visits, there is no information about the changing status of the patient’s condition, adherence to treatment, or the response to it. In recent years, there has been a digital transformation in health care models, and these new technologies could provide aids to measure, monitor, and treat frailty in the older population to prevent health adverse events. Different studies have been performed within the framework of information and communication technologies and frailty treatment and prevention, but the results obtained are not conclusive, given that the technologies used are very different, and the results are very diverse [7,8].

With this approach in mind, we developed the FACET (Frailty Care and Well Function) technological ecosystem to improve the effectiveness of management of frail patients. A technological ecosystem refers to a complex network of interrelated technologies, services, and stakeholders that interact and depend on each other to create a unified and functional environment. This system automatically collects information with high predictive power for adverse events at home. This information comprises speed, power in the lower limbs, and involuntary weight loss. This valuable information is complemented with questionnaires to assess nutritional and other data about the functional status, which are uploaded to the platform. Data are provided to the geriatric team through the FACET technological ecosystem.

This research work aims to evaluate the impact of the FACET technological ecosystem when supporting a comprehensive geriatric intervention and follow-up. To do so, a randomized pilot study was designed to assess whether the information provided by the remote sensors helps early detection of functional changes, thereby promoting an adjusted multimodal intervention in prefrail and frail older adults compared to usual care during a 6-month period.

## Methods

### Trial Design

This is a multicenter, randomized, simple blind intervention study, with a duration of 11 months (5 months for recruitment and 6 for intervention). This study was conducted from August 2018 to June 2019 in the geriatric outpatient clinics of 2 Spanish hospitals (Getafe University Hospital and Albacete University Hospital). This study was registered in ClinicalTrials.gov (NCT03707145).

### Participants and Randomization

#### Participant Recruitment

Participants were recruited in-person and by telephone from several settings, including the geriatric outpatient clinic, acute care unit (after discharge), and primary care outpatient clinics. Initially, a prescreening interview was performed in the outpatient geriatric facility to identify potentially eligible patients who met the following inclusion criteria: (1) age ≥70 years, (2) living at home, (3) having a caregiver or supervision at home, (4) Barthel index ≥90, (5) having at least 4 comorbidities, (5) prefrail older adults meeting 1 or 2 Fried frailty phenotype criteria, and (6) frail older adults meeting 3, 4, or 5 Fried frailty phenotype criteria.

The exclusion criteria were (1) inadequate home infrastructure to host the required technology, (2) inability to understand how to use the FACET system, (3) illness that impedes performing the prescribed therapy or follow-up (acute myocardial infarction in the last 3 months, unstable cardiovascular disease, terminal disease of <12 months of life expectancy, other pathologies involving clinical instability), (4) alcohol/drug abuse, (5) living with a participant in the trial, and (6) participation in other interventional clinical studies at the same time. Finally, to determine if a participant could or could not understand and use the FACET system, the following procedure was performed:

1. Provide indications on how to react to a reminder to perform an action through a mobile device of identical conditions to the one that will be used during the experimentation.
2. Provide indications on how to fill out a test with the mobile device.
3. Generation of a reminder to fill out a test different from the previously shown.

If the potential participant reacted to the alarm and completed the test by him/herself, he/she was considered a suitable candidate. Once it was verified that the participants met the eligibility criteria, the informed consent was signed, and then they were randomized to the intervention or the control group.

#### Settings and Locations Where the Data Were Collected

Recruitment, assessment, follow-up, and treatment were performed in parallel in 2 institutions: Getafe University Hospital and Albacete University Hospital. Researchers in charge of performing the intervention or collecting the data along with the follow-up were different. Although information gathered by the FACET technological ecosystem was revised every week by a nonblinded geriatrician, who after reviewing it, designed and performed the different needed interventions during the development of the study in the intervention group, data about the outcomes were collected through face-to-face interviews by another geriatrician who was blinded regarding the branch where the participant had been randomly allocated every 3 months.

### Outcomes

#### Primary Outcomes

The primary outcomes of this study were as follows: (1) to assess whether the FACET monitoring system prevents the worsening of frailty status according to the Frailty Trait Scale-5 items (FTS-5) (Table S1 in Multimedia Appendix 1) and the Fried frailty phenotype criteria (Table S2 in Multimedia Appendix 1) at 3 and 6 months and (2) to assess whether the FACET monitoring system improves frailty status according to FTS-5 and the Fried frailty phenotype criteria at 3 and 6 months. The outcomes were assessed through the following criteria: frailty worsening and improvement.

#### Assessment of Primary Outcomes

##### Frailty Worsening

The worsening in frailty status was evaluated according to 2 different criteria: (1) changes in the score in FTS-5 (worsening of 2.5 points or more) [9] and worsening by 1 criterion according to Fried frailty phenotype criteria [10] and (2) by analyzing transitions from nonfrail to frail according to FTS-5 [11] and from prefrail to frail when Fried frailty phenotype criteria were used [12]. Measurements were performed at baseline, 3 months, and 6 months of follow-up.

##### Frailty Improvement

Improvements in frailty were quantified according to 2 sets of criteria: (1) within FTS-5, based on an improvement of 2.5 points or more [9] and decrease by at least 1 criterion of the Fried frailty phenotype criteria [10] and (2) analyzing transitions from frail to nonfrail according to FTS-5 [11] and either from frail to prefrail or robust or from prefrail to robust according to Fried frailty phenotype criteria [12].

#### Secondary Outcomes

The secondary outcomes were as follows:

1. Changes in quality of life assessed using EQ-5D-5L at the initial visit, at 3 months, and after 6 months of follow-up.
2. Changes in the use of health resources assessed by open questions on number of visits to the emergency room, number of visits to the primary care physician, number of visits to nursing staff, number of visits to primary care, number of falls, and hospital admissions in the last 6 months. These data were collected at the initial visit and after 6 months of follow-up.

### Hypothesis

The combination of information and communication technologies improves the monitoring of the patients at home with an impact on the frailty status.

### Variables

Data were collected from participants during face-to-face visits in outpatient clinics at baseline, 3 months, and 6 months, except for the cognitive test that was assessed at baseline and at 6 months. The following data were collected: (1) demographic data, (2) comorbidities, (3) medication, (4) Barthel index [13], (5) frailty phenotype criteria [12], (6) FTS-5 [11], (7) gait speed in 6 meters [14], (8) Mini Mental Status Examination [15], (9) EQ-5D-5L [16], and (10) use of health resources in the last 6 months, that is, number of emergency visits, number of hospital admissions, number of visits to primary care physician, number of visits to nursing staff, number of visits to specialist physician, and number of falls.

### Technological Ecosystem Description

#### Overview of the FACET Monitoring System

**Figure 1:**
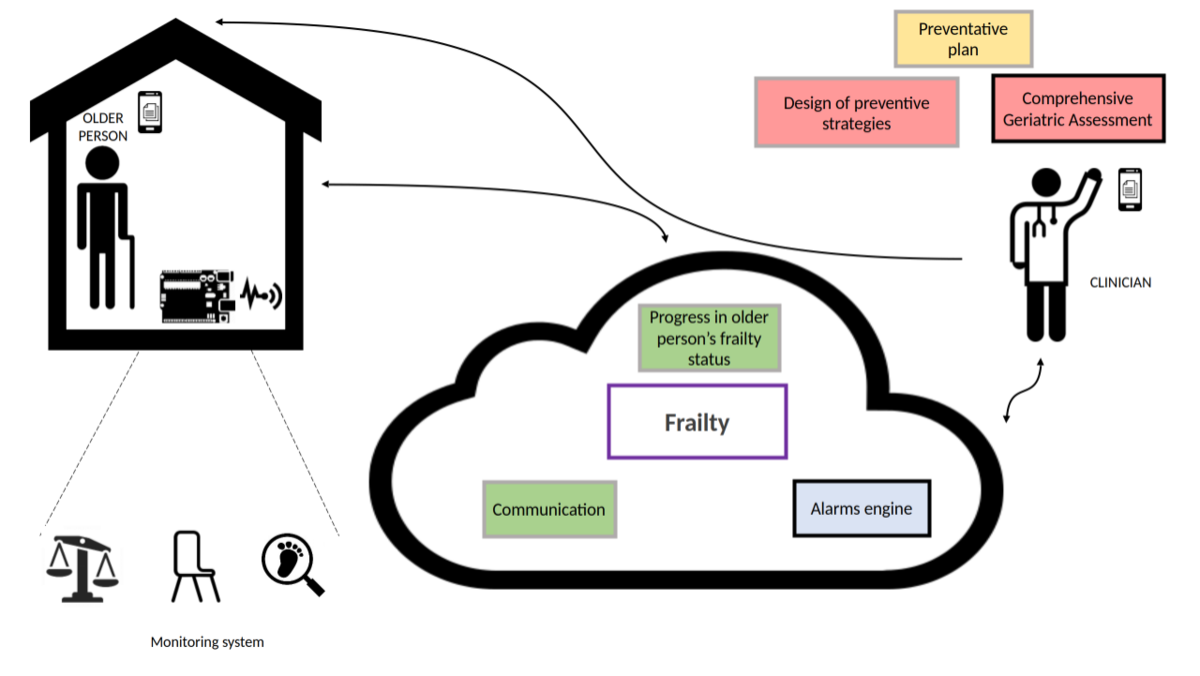
The FACET (Frailty Care and Well Function) system: components and users.

The FACET monitoring system is a technological ecosystem developed jointly by the Getafe University Hospital and Universidad Politécnica of Madrid that lies on the technological substrate of a Europe Union-funded (European Institute of Innovation and Technology-Health) research and innovation project: FACET. This project produced a mature and low-cost home monitoring system that aims at preventing functional decline among prefrail and frail older adults by detecting early functional changes and by generating alerts to promote early interventions to prevent potential adverse events that may lead to disability and dependency. Furthermore, the FACET technological ecosystem provides a means to interconnect the geriatric care team with older adults (see Figure 1). It is important to remark that the technology under study was fully developed following a cocreation approach [17,18], integrating knowledge and experience from all relevant actors in the process: older adults, health care professionals, informal caregivers, and technology experts.

#### FACET Monitoring System

The FACET technology has 2 different components (Figure 1): (1) home monitoring subsystem for patients and (2) web interface for professionals.

#### Home Monitoring Subsystem for Patients

### Mobile App for Older Adults

**Figure 2:**
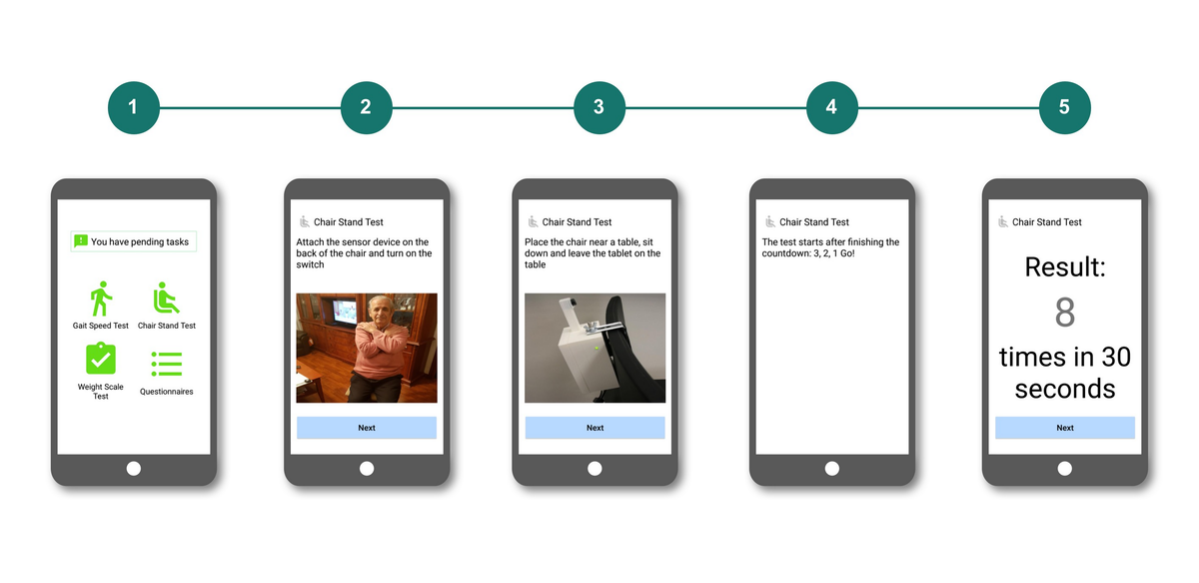
Mobile app in this study.

The mobile app (Figure 2) in the monitoring subsystem offers the following functionalities: continuous frailty follow-up using a home monitoring kit that produces information (gait speed, power in the lower limbs, involuntary weight loss) that is later processed to trigger potential deterioration alerts [19,20]; access to a customized therapeutic multicomponent intervention provided by the nonblinded geriatrician (medical treatment, VIVIFRAIL physical activity program [21], and nutritional recommendations); retrieving their own data on usage; communication with a geriatrician via asynchronous channels; notiﬁcations on pertinent alarms related to health to activate early interventions, with the goal of preventing disability; and reminders to perform the tests that must be performed at home. The participants in the intervention group accessed the mobile app through a tablet with 4G connection, provided by the study.

### Monitoring System

**Figure 3:**
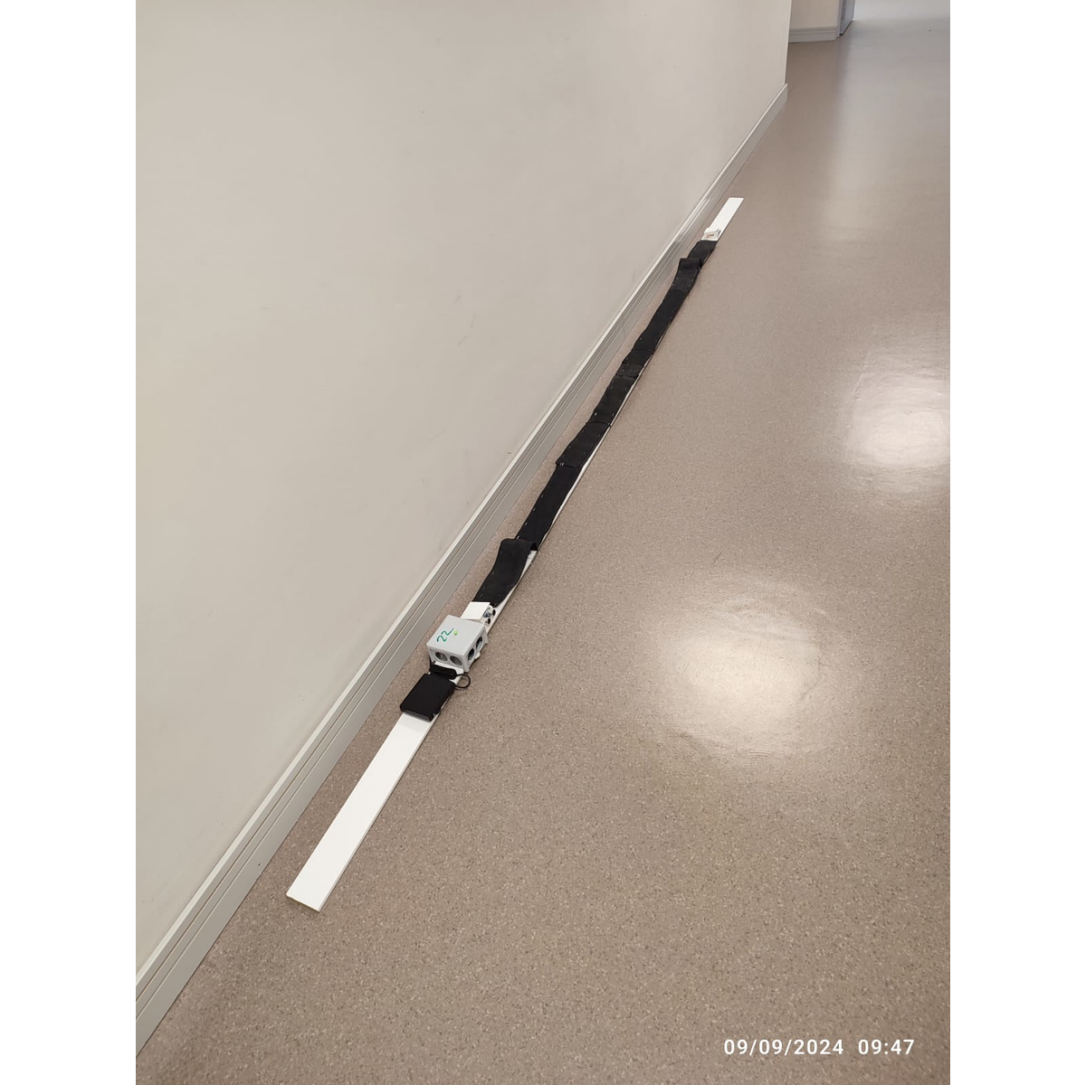
Gait speed sensor.

**Figure 4:**
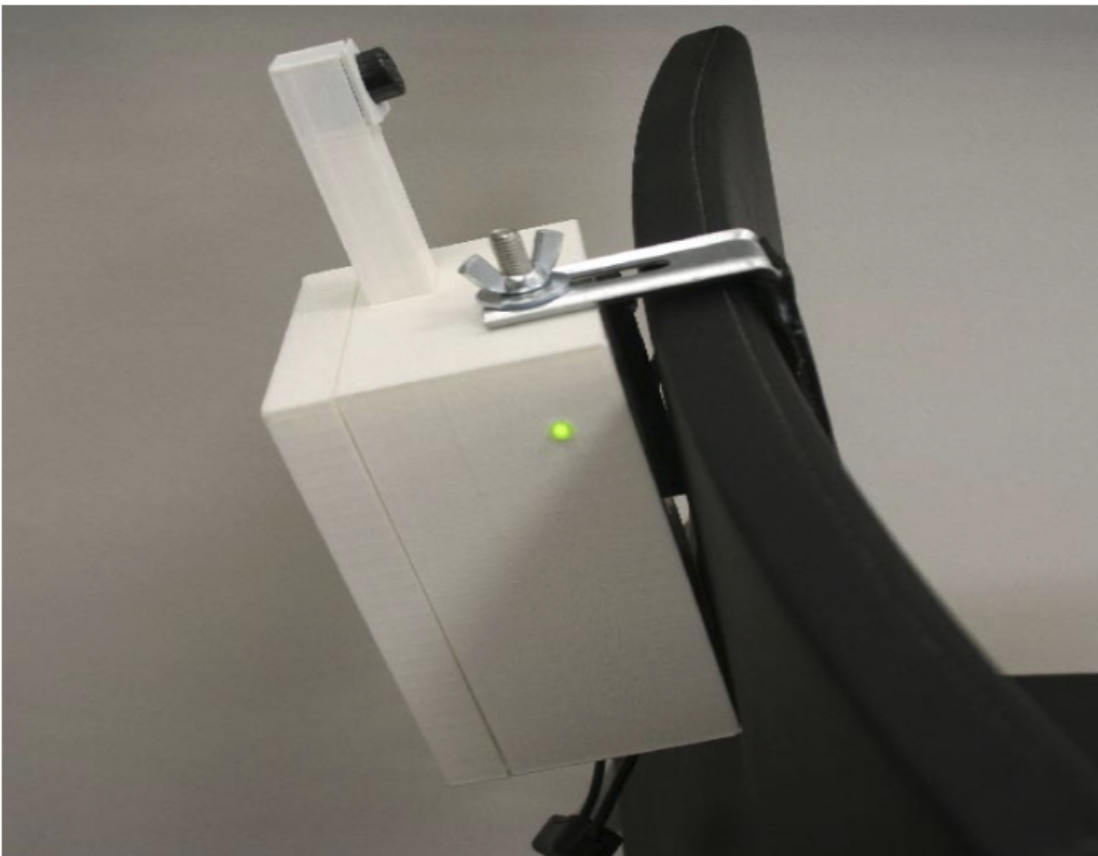
Lower limbs power sensor.

The interaction with the home monitoring subsystem is handled by a mobile app that acts as a guiding element to the older person, as a data concentrator (Bluetooth connection with the monitoring kit) (Figure 1) and as data input point, not only enabling the older adults using the sensors but also completing a set of questionnaires to enrich the information handled by the clinical professionals. The home monitoring kit has been designed to measure variables with high predictive values for adverse events. This kit consists of a gait-speed sensor (Figure 3) [20], a sensor to indirectly (through the chair stand test) measure power in the lower limbs (Figure 4) [19], and a wireless commercial weight scale to measure involuntary weight loss. This information was enriched to build a short comprehensive geriatric assessment thought different questionnaires, which were tailored to language appropriate for the study’s target population. The questionnaires that patients complete through the monitoring system is based on the Fried frailty phenotype criteria [12], Mini Nutritional Assessment [22], and Barthel index [23]. Information collected by this home monitoring kit is processed to trigger potential deterioration alarms.

#### Web Interface for Professionals

The web interface provides essential infrastructure to offer functionalities to health care professionals. These functionalities include storing and accessing clinical information, asynchronous messaging between patients and the geriatrician team, displaying alarms based on patient monitoring results, tracking patient’s clinical progress (questionnaires, functional test, etc), as well as prescribing and modifying treatments.

#### Sample Size

There are no similar studies allowing for a priori estimation of the necessary sample size. Therefore, an initial recruitment target of 90 participants was set based on the following criteria:

1. There are 2 main groups of interest: frail and prefrail individuals.
2. Based on commonly used standards, the recruitment goal is set at 20 participants per group of interest, totaling 40 participants for the control group and 40 for the intervention group.
3. Assuming a 10%-15% loss from the above estimation, the target sample size is established at 90 participants (N=90).

#### Randomization

For participant allocation into either the control or the intervention group, a stratified randomization by age (70-85 years, >85 years), sex (male/female), diagnosis (frail and prefrail among Fried frailty phenotype criteria), and educational level (higher education, illiterate, others) was performed to ensure that the 2 research arms were properly balanced. Participants were allocated using the MINIM tool [24] configured according to the study needs. Randomization was performed by the team members not directly involved in the development of the clinical trial. A recruitment target of 90 participants was established: 44 participants were allocated to the control group and 46 to the intervention group.

Three professional roles were involved in this study:

1. Nonblinded geriatricians to check and verify participant’s eligibility based on the inclusion and exclusion criteria, explain the study details and obtain informed consent before randomization, monitor progress through the FACET system, and adjust the treatment as needed along the study.
2. Blinded geriatricians who participated in the prerandomization tasks of the study. They did not receive information about the arm to which the patient was randomly allocated. They also recorded the participant’s evaluations at the baseline visit, 3 months, and 6 months. These researchers did not have access to the data from the FACET monitoring system.
3. Biomedical engineers conducted training sessions with the participants regarding the use of the FACET monitoring system, installation of technology at home, and resolution of technical problems during the development of the study.

#### Interventions

After signing the informed consent, the participants were allocated into the control or the intervention group. The control group (n=44) received usual geriatric care through classical ways (comprehensive geriatric assessment; adjustment of polypharmacy; physical, cognitive, and nutritional prescription done face-to-face in classical patient visits). Participants in the intervention group (n=46) received the usual health care by a geriatric team, but they were supported by the information provided by the FACET monitoring system.

Participants were assessed to collect the variables previously described at baseline and after 3 and 6 months of follow-up. After the baseline evaluation, a treatment plan was designed with the following core components: medication adjustment, prescription of physical exercise based on the VIVIFRAIL guide [21], and dietary recommendations. In the intervention group, the participants were periodically and remotely supervised by their nonblinded geriatrician who scheduled the questionnaires and tests that the participants must perform at home to monitor the progress in their conditions. Frailty scale, chair stand test, gait speed (2.4 meters), and weight measurements were performed once weekly. Barthel index measurements and frequently asked questions were administered once every 2 weeks, and the Mini Nutritional Assessment-Short Form was administered once monthly. These sets of information were captured by the system and stored in the project-dedicated server. The system provides alerts to the clinician (nonblinded geriatrician) when pre-established changes were detected.

Nonblinded geriatricians checked the platform daily to see if any alarm had been generated. In case an impairment was detected, the geriatrician would call by phone to check the patient’s health status and assess them. The nonblinded geriatrician, after this phone call, could provide an appointment to meet the patient face-to face if needed. When clinically indicated, he/she made changes to the treatment. Moreover, the participants and the nonblinded researcher could contact through a basic asynchronous communication module along the study. Those in the control group followed the usual guidelines without technological monitoring.

#### Technological Revisions and Updating

The technological solution was frozen during the intervention, which only fixed malfunctions without interfering with the functionalities and services provided.

#### Statistical Analysis

Statistical analysis was performed on an intention-to-treat basis. Summary statistics were presented as mean (SD) and n (%). Mann-Whitney and chi-square tests were performed to verify the hypothesis of the randomization scheme. To assess the main and secondary objectives, we compared both interventions (with and without technology) by using multivariate linear regression models for continuous response, multivariate logistic models for dichotomous response (improvement and worsening events), and Poisson models for count responses (ie, visits to the doctor). The analysis was adjusted for basal functional status (FTS-5) and gender. All the analyses were performed using R software (R Foundation for Statistical Computing) for Windows version 4.1.2. Statistical significance was set at *P*<.05.

#### Ethics Approval

This study followed the principles of the Declaration of Helsinki and was approved by the Getafe Hospital and Albacete Hospital Clinical Research ethics committees. This study was approved by the Getafe Hospital Ethics Committee (PY: 17/85). All participants provided written informed consent before screening (Multimedia Appendix 2). Participants’ data were anonymized, and data correspondence was stored in a secure digital file supervised by a study engineer. There was no financial compensation.

## Results

### Participant Recruitment

**Figure 5:**
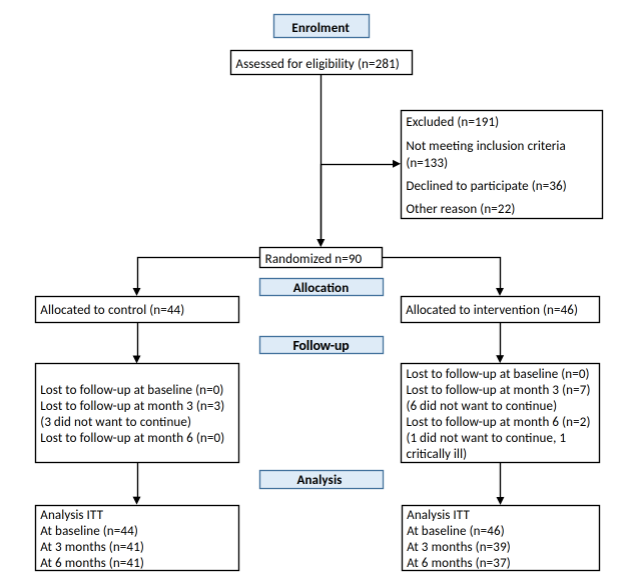
CONSORT (Consolidated Standards of Reporting Trials) flowchart diagram. ITT: intention-to-treat.

Of the 281 individuals who were initially evaluated, 191 were excluded because they did not meet the inclusion criteria (n=133), they refused to participate in the study (n=36), and of other reasons (n=22). Recruitment ceased when 90 participants were enrolled. Randomization resulted in the allocation of 44 participants to the control group and 46 participants to the intervention group. In the third month of follow-up, 3 participants from the control group and 7 in the intervention group refused to continue in the study. In the follow-up at 6 months, 2 participants from the intervention group dropped out. Finally, from the original sample, 41 participants in the control group and 37 in the intervention group completed the full study (Figure 5).

### Baseline Data

The baseline characteristics of the participants are summarized in Table 1. Both groups exhibited similar baseline traits. The mean age of our study population was 82.33 (SD 5.91) years. The majority were women (65/90, 72%), had limited education (either no education or only primary education), and did not use technology daily. Participants in both groups showed independence in basic activities of daily living, with a mean Barthel index of 94.11 (SD 3.72). Participants met a mean of 2.73 (SD 0.86) criteria, and 50% (45/90) were categorized as frail and 50% (45/90) as prefrail. The mean score of FTS-5 was 22.89 (SD 6.14). The Charlson index data indicated that our patients exhibited high levels of comorbidity. Additionally, the recruited patients were characterized by polypharmacy. Data on functional status, gait speed, Short Physical Performance Battery, and Timed Up and Go indicated mild levels of physical impairment. The mean cognitive status values indicated very mild cognitive impairment. No significant differences were observed between the 2 groups, confirming the adequacy of the randomization process.

**Table 1:** Overall group characteristics.

			Total (N=90)		Intervention group (n=46)		Control group (n=44)		*P* value	
Age (years), mean (SD)			82.33 (5.91)		82.11 (5.42)		82.56 (6.43)		.77	
Males, n (%)			25 (28)		14 (30)		11 (25)		.56	
**Studies, n (%)**										.14
	No education	32 (36)		20 (43)		12 (28)				
	Primary education	43 (48)		20 (43)		23 (53)				
	Secondary education	11 (12)		5 (11)		6 (14)				
	Tertiary education	3 (43)		1 (32)		2 (55)				
**Experience with technology, n (%)**										.32
	Never	56 (63)		30 (65)		26 (60)				
	1-2 times	5 (66)		4 (999)		1 (32)				
	Occasional use	6 (77)		3 (77)		3 (77)				
	Daily use	22 (25)		9 (20)		13 (30)				
Charlson index, mean (SD)			5.43 (1.83)		5.64 (1.93)		5.17 (1.69)		.31	
Number of drugs, mean (SD)			9.44 (3.74)		9.91 (3.91)		8.95 (3.58)		.40	
Mini Mental State Examination, mean (SD)			26.81 (3.04)		26.98 (2.68)		26.64 (3.39)		.93	
Mini Nutritional Assessment, mean (SD)			24.63 (3.73)		24.49 (3.82)		24.78 (3.66)		.74	
Barthel index, mean (SD)			94.11 (3.72)		94.24 (3.94)		93.98 (3.51)		.81	
Short Physical Performance Battery, mean (SD)			7.21 (2.54)		7.41 (2.36)		7.0 (2.72)		.46	
Gait speed 6 m, mean (SD)			0.71 (0.22)		0.72 (0.25)		0.69 (0.2)		.92	
**Frailty phenotype criteria**										
	Number of criteria, mean (SD)	2.73 (0.86)		2.74 (0.80)		2.73 (0.92)		.84		
	Prefrail, n (%)	45 (50)		22 (48)		23 (52)		.67		
	Frail, n (%)	45 (50)		24 (52)		21 (48)		.67		
**Frailty Trait Scale-5 items**										
	Mean score, mean (SD)	22.89 (6.14)		23.40 (6.56)		22.38 (5.72)		.64		
	Frail, n (%)	27 (30)		14 (32)		13 (29)		.82		
	Nonfrail, n (%)	63 (70)		32 (68)		31 (71)		.82		
EQ-5D-5L, mean (SD)			56.07 (25.3)		54.15 (24.78)		58.07 (25.92)		.36	

### Primary Outcomes and Estimation

#### Frailty Worsening

##### Changes in FTS-5 Score and Worsening of 1 criterion in the Fried Frailty Phenotype Criteria

When we compared the intervention and control groups along the time through changes in FTS-5, we observed a 77% reduction in the risk of deterioration at the limits of statistical significance (odds ratio [OR] 0.23, 95% CI 0.05-1.09; *P*=.06) at 3 months in the intervention group that reached statistical significance at 6 months of follow-up, with a 74% reduction in the risk of deterioration (OR 0.26, 95% CI 0.07-0.98; *P*=.04) (Table 2). When analyzing data based on 1-point worsening according to the Fried frailty phenotype criteria, the results showed that the intervention group had a 92% lower likelihood of worsening compared to that in the control group at 6 months of follow-up (OR 0.08, 95% CI 0.01-0.67; *P*=.02) (Table 2). As there were no events at 3 months in any of the 2 groups, it was not possible to assess the effect.

**Table 2:** Frailty worsening assessed by the Frailty Trait Scale-5 items (worsening of 2.5 points) and changes in 1 point in the Fried frailty phenotype criteria.

		Odds ratio (95% CI)	*P* value
**Frailty Trait Scale-5 items**			
	Month 0-month 3	0.23 (0.05-1.09)	.06
	Month 0-month 6	0.26 (0.07-0.98)	.04
**Fried Frailty Criteria**			
	Month 0-month 3	None^a^	—^b^
	Month 0-month 6	0.08 (0.01-0.67)	.02

^a^The frailty status of none of the participants worsened (Fried criteria) at 3 months of follow-up.

^b^Not available.

##### Transitions From Nonfrail to Frail According to FTS-5 and From Prefrail to Frail by Fried Frailty Phenotype Criteria

We did not find significant changes in frailty transitions at either 3 months or 6 months on either of the 2 scales (see Table 3).

**Table 3:** Transitions from nonfrail to frail by Frailty Trait Scale-5 items and transition from prefrail to frail by Fried frailty phenotype criteria.

		Odds ratio (95% CI)	*P* value
**Frailty Trait Scale-5 items**			
	Month 0-month 3	0.52 (0.16-1.66)	.27
	Month 0-month 6	0.76 (0.24-2.42)	.64
**Fried Frailty Criteria**			
	Month 0-month 3	None^a^	—^b^
	Month 0-month 6	0.43 (0.03-5.74)	.52

^a^The frailty status of none of the participants worsened (Fried criteria) at 3 months of follow-up.

^b^Not available.

#### Frailty Improvement

##### Frailty Status

There was a higher likelihood of improvement in frailty status through FTS-5 in the intervention group compared to the control group at 3 months, with an OR of 4.16 (95% CI 1.57-11.03; *P*=.004). The benefits were retained for 6 months, with an OR of 2.63 (95% CI 1.004-6.90; *P*=.047). According to Fried frailty phenotype criteria, there was an improvement in the intervention group compared to the control group, with an OR of 4.50 (95% CI 1.17-17.38; *P*=.03) at 3 months of follow-up. The improvement was not observed at the 6-month follow-up (Table 4).

**Table 4:** Frailty improvement assessed by changes in 2.5 points in Frailty Trait Scale-5 items and changes in 1 criterion by Fried frailty phenotype criteria.

		Odds ratio (95% CI)	*P* value
**Frailty Trait Scale-5 items**			
	Month 0-month 3	4.16 (1.57-11.03)	.004
	Month 0-month 6	2.63 (1.004-6.90)	.047
**Fried Frailty Criteria**			
	Month 0-month 3	4.50 (1.17-17.38)	.03
	Month 0-month 6	1.37 (0.37-5.03)	.63

##### Transitions From Frail to Nonfrail According to FTS-5 and From Prefrail to Robust or From Frail to Prefrail or Robust by Fried Frailty Phenotype Criteria

We did not observe statistically significant results in FTS-5 at 3 or 6 months of follow-up in the transitions from frail to nonfrail according to FTS-5 and from prefrail to robust or from frail to prefrail or robust by the Fried frailty phenotype criteria. However, when we analyzed transitions by the Fried frailty phenotype criteria, the results indicated a marginal higher likelihood of improvement in frailty status occurring in the intervention group compared to that in the control group, with an OR of 3.10 (95% CI 1.01-9.54; *P*=.049). These benefits did not persist at 6 months, with an OR of 1.50 (95% CI 0.54-4.13; *P*=.44) (Table 5).

These transitions are shown in more detail in Table 6 (FTS-5) and Table 7 (Fried frailty criteria).

**Table 5:** Transitions from frail to nonfrail by Frailty Trait Scale-5 items and transitions from prefrail to robust or from frail to prefrail or robust by Fried frailty phenotype criteria.

		Odds ratio (95% CI)	*P* value
**Frailty Trait Scale-5 items**			
	Month 0-month 3	1.93 (0.60-6.21)	.27
	Month 0-month 6	1.32 (0.41-4-19)	.64
**Fried Frailty Criteria**			
	Month 0-month 3	3.10 (1.01-9.54)	.049
	Month 0-month 6	1.50 (0.54-4.13)	.44

**Table 6:** Frailty transitions, as shown by Frailty Trait Scale-5 items.

			Worsening, n (%)		No changes, n (%)		Improving, n (%)
**Month 0-month 3**							
	Control (n=41)	8 (20)		19 (46)		14 (34)	
	Intervention (n=39)	2 (5)		15 (39)		22 (56)	
**Month 0-month 6**							
	Control (n=41)	9 (22)		13 (32)		19 (46)	
	Intervention (n=37)	3 (8)		10 (27)		24 (65)	

**Table 7:** Frailty transitions, as shown by the Fried frailty phenotype criteria.

			Robust, n (%)	Prefrail, n (%)	Frail, n (%)
**Month 0-month 3**					
	**Robust**				
		Control (n=0)	0 (0)	0 (0)	0 (0)
		Intervention (n=0)	0 (0)	0 (0)	0 (0)
	**Prefrail**				
		Control (n=22)	2 (9)	20 (91)	0
		Intervention (n=18)	2 (11)	16 (89)	0 (0)
	**Frail**				
		Control (n=19)	0 (0)	7 (37)	12 (63)
		Intervention (n=21)	1 (5)	14 (67)	6 (29)
**Month 0-month 6**					
	**Robust**				
		Control (n=0)	0 (0)	0 (0)	0 (0)
		Intervention (n=0)	0 (0)	0 (0)	0 (0)
	**Prefrail**				
		Control (n=22)	3 (14)	17 (77)	2 (9)
		Intervention (n=19)	4 (21)	14 (74)	1 (5)
	**Frail**				
		Control (n=19)	0 (0)	9 (47)	10 (53)
		Intervention (n=18)	1 (6)	9 (50)	8 (44)

In this same regard, as a whole, the mean improvement in Fried’s criteria was marginally higher in the intervention group at 3 months (OR 0.34, 95% CI –0.05 to 0.74; *P*=.09) and 6 months (OR 0.42, 95% CI –0.02 to 0.87; *P*=.06), while it reached statistical significance when FTS-5 was used, both at 3 months (OR 2.85, 95% CI 0.92-4.77; *P*=.005) and 6 months (OR 2.10, 95% CI 0.07-4.14; *P*=.04) (Table S3 in Multimedia Appendix 1).

### Secondary Outcomes

We did not find significant changes in any of the secondary outcomes, except for falls. We did not detect changes in the visits to the emergency room, hospitalizations, visits to primary care physician and nurse, number of falls, or quality of life (Table S4 in Multimedia Appendix 1).

## Discussion

### Principal Findings

The main finding of our study is that the use of the FACET technological ecosystem over a 6-month period prevents impairment in older adults and improves their frailty status, irrespective of the method used to assess it, and prevents the progression of frailty according to FTS-5. Additionally, our data suggest that this benefit is observed quite early with the use of the FACET technological ecosystem, and it is maintained after 6 months of follow-up, although with a tendency to moderate its impact.

FACET per se does not provide any intervention (changes in physical exercise, nutrition, drugs, etc), which is decided by the geriatrician, but only monitors the functional variables. Therefore, intensive monitoring that allows early detection of functional deterioration by generating alerts to the geriatrician is useful for obtaining benefits in this prefrail and frail population, no matter the type of intervention provided. This finding reinforces the concept of a low functional reserve classically linked to the presence of frailty [6]—a fact that highlights the need of an intervention as early as possible to avoid further deteriorations that are quickly developed in the absence of any intervention.

Although data are consistent across frailty scales, FTS-5 shows the most reliable data. FTS-5 assesses a continuous multisystemic gradient, which makes it a more sensitive scale to changes and enables better detection of biological dysfunction, ranging from identifying the most robust to the most vulnerable individuals [11,25]. FTS-5 exhibits a heightened sensitivity in identifying even the slightest alterations in frailty status, which could justify the results obtained. FTS-5 and Fried frailty phenotype criteria show a low agreement, as recently reported in a study that evaluated the different tools that assess frailty [26], raising the possibility that they capture different dimensions of frailty [27,28]. In this regard, some researchers postulate the existence of different types of frailty [27].

### Comparison With Prior Works

All previous studies [8,29,30] conducted in this field differ from ours in their inability to integrate information acquired through novel technologies with comprehensive geriatric assessments, aiming to enhance the evaluation and intervention processes for frailty. Numerous clinical studies [8,31-33] have been performed to enhance frailty diagnosis and treatment through information and communication technologies. However, results from these studies vary significantly, reflecting a wide array of devices employed for these purposes. This diversity complicates drawing definitive conclusions about the practical use and implementation of new technologies in clinical settings [8].

Most studies [33-35] assess frailty based on the frailty phenotype, while others [36] employ the frailty index. A study similar to ours was conducted in the United States by Upatising et al [29], involving 205 older adults (100 men) with an average age of 80.4 years, recruited from primary and community care. That study aimed to assess whether 12-month home monitoring could prevent frailty and mortality in patients with clinical issues. The devices utilized included a remote surveillance system, a health guide placed in the home, and other peripheral equipment connected to the health care system. Parameters such as heart rate, blood pressure, oxygen saturation, glucose level, and weight were monitored at home. Results showed that home monitoring did not reduce the functional decline, as measured by frailty and mortality rates, among older adults. Moreover, the home monitoring system itself did not induce changes in patients’ functional status. Instead, the study indicated a need for re-evaluating the organizational model. A major limitation of that study was the absence of a clinical intervention, as they only detect status that did not promote an intervention, while in our study, the detection of an impairment promoted an alert motivating a contact between the clinician and the patient. This fact may at least partially explain the differences in our findings. In fact, simply monitoring should not be expected to produce any effect without intervention.

Another randomized clinical trial conducted by Geraedts et al [31] investigated whether frail older individuals using a portable physical activity sensor showed increased adherence and efficacy in a personally tailored home-based physical activity program. Feedback was provided to the participants, including videos demonstrating the exercises. That study concluded that patients using these sensors exhibited superior mobility outcomes compared to the control group.

### Strengths and Limitations

This study has both strengths and limitations. One of its primary strengths lies in its internal validity, given its status as a randomized controlled trial. The control and intervention groups were meticulously balanced and comparable at the baseline, enhancing the study’s credibility. Moreover, this study stands as the first of its kind to assess the effectiveness of an intervention integrating a home monitoring system with a classically provided comprehensive geriatric assessment and intervention. This assessment evaluates cognitive, affective, and physical domains; nutritional status; and social circumstances of older individuals, while also attempting to reshape the existing organizational model. The participants were older adults, which provides the study with validity when implementing the results in clinical practice. Another strength lies in the cocreation of the technology with older individuals, ensuring high adherence to the FACET technological ecosystem [18].

However, our study is not without its limitations. There were several dropouts in the intervention group, potentially attributed to the low digital literacy of the participants, who might have felt overwhelmed by the technology. Another limitation stemmed from the challenge of maintaining blinding throughout the study. Some participants revealed their research group affiliation during evaluation visits, although the researcher remained unaware of the previous assessments.

### Conclusion

Our findings demonstrate that the FACET technological ecosystem effectively helps in the early identification of changes in the functional status of prefrail and frail older adults, facilitating prompt clinical interventions, thereby improving health outcomes in terms of frailty and functional status, which may prevent disability and dependency. We believe that the integration of information and communication technologies such the one used in this study is an asset in routine clinical practice. Nevertheless, further studies are warranted to advance our understanding in this area.

## Appendix

Multimedia Appendix 1 Supplementary data.

Multimedia Appendix 2 Informed consent form.

Multimedia Appendix 3 CONSORT eHEALTH checklist (V.1.6.2).

## Abbreviations

FACET: Frailty Care and Well Function
FTS-5: Frailty Trait Scale-5 items
OR: odds ratio

## Data Availability

Restrictions apply to the availability of the data used in this study due to the private nature of the health information contained. These data are not publicly available but are available from the authors upon reasonable request and with permission of Getafe University Hospital.
