# Supplementary material for: Effects of Monitoring Frailty Through a Mobile/Web-Based Application and a Sensor Kit to Prevent Functional Decline in Frail and Prefrail Older Adults: FACET (Frailty Care and Well Function) Pilot Randomized Controlled Trial
Source: J Med Internet Res. 2024 Oct 22;26:e58312. doi: 10.2196/58312 (PMC11538877; doi:10.2196/58312)
Supplement: Multimedia Appendix 1 [file jmir_v26i1e58312_app1.docx]

# APPENDIX 1: ADDITIONAL MATERIAL

*Table S1. Frailty Trait Scale-5.*

| **Score** | **BMI, kg/m^2 a^** | | **PASE^b^** | **Gait speed^3^** | **Grip strength, kg** | | **Score** | **Progressive Romberg** | |
| --- | --- | --- | --- | --- | --- | --- | --- | --- | --- |
|  |  |  |  |  | **women** | **men** |  | **Position** | **Seconds** |
| **0** | 23.01-26.99 |  | >194 | <2.45 | >22 | >29 | 0 | Tandem | ≥10 |
| **1** | 27-28.99 | 21.01-23 | 174.61-194 | 2.45-2.99 | 19.81-22 | 26.11-29 | 2.5 | Trandem | 3.01-9.99 |
| **2** | 29-30.99 | 19.01-21 | 155.21-174.6 | 3.00-3.54 | 17.61-19.8 | 23.21-26.1 | 5 | TAndem | ≤3 |
| **3** | 31-32.99 | 17.01-19 | 135.81-155.2 | 3.55-4.09 | 15.41-17.6 | 20.31-23.2 |  | Semi-tandem | ≥10 |
| **4** | 33-34.99 | 15.01-17 | 116.41-135.8 | 4.10-4.64 | 13.21-15.4 | 17.41-20.3 | 7.5 | Semi-tandem | <10 |
| **5** | 35-36.99 | 13.01-15 | 97.01-116.4 | 4.65-5.19 | 11.01-13.2 | 14.51-17.4 |  | Side by side | ≥10 |
| **6** | 37-38.99 | 11.01-13 | 77.61-97 | 5.20-5.74 | 8.81-11.0 | 11.61-14.5 | 10 | Side by side | <10 |
| **7** | 39-40.99 | NA | 58.21-77.6 | 5.75-6.29 | 6.61-8.8 | 8.71-11.6 |  |  |  |
| **8** | 41-42.99 | NA | 38.81-58.2 | 6.30-6.84 | 4.41-6.6 | 5.81-8.7 |  |  |  |
| **9** | 43-44.99 | NA | 19.41-38.8 | 6.85-7.39 | 2.21-4.4 | 2.91-5.8 |  |  |  |
| **10** | ≥45 | NA | 0-19.4 | ≥7.4 | 0-2.2 | 0-2.9 |  |  |  |

a: Body mass index

b: Physical Activity Scale for the Elderly.

c: Gait speed refers to time in accomplish 3-metres at usual pace.

FTS5 includes all the items of the table (range 0-50), and frail participants are those with FTS5 scores >25.

| 1. **Weight Loss:**   Ask the patient if they have lost more than 4.5 kg of weight involuntarily in the past year. | Yes | No |
| --- | --- | --- |
| **2- Exahustion:**  1º I felt that everything I did was an effort during the past week  2º Last week, I felt I could not go on  If either of the two responses to the previous questions is YES, the score will be 1. If in both cases it were NO, the score will be 0. | Yes | No |
| 1. **Physical activity:**   Does the patient engage in weekly physical activity?: Men: <383 kcal/week (similar to walking <2 hours and 30 minutes / week) Women: <270 kcal/week (similar to walking <2 hours / week) | Yes | No |
| 1. **Slowness**:   Measured as the time it takes for the patient to walk 4.5 meters at their usual walking pace.  MEN  ≤173 cm >7Seg  >173 cm >= 6seg  WOMEN  ≤159 cm >7Seg  >159 cm >6Seg | Yes | No |
| **5- Weakness Grip strength (Kg):**  Determine if the patient has weakness based on BMI and gender. Example: A man with a grip strength of 28 kg and BMI of 26 should be classified as weak  il  IMC ♂CUT-OFF IMC ♀ CUT-OFF  ≤ 24 <29 kg ≤ 23 <17 kg  24,1-26 <30 kg 23,1-26 <17,3 kg  26,1-28 <30 kg 26,1-29 <18 kg  >28 <32 kg >29 <21 kg | Yes | No |

*Table S2. Fried Frailty Phenotype*

Fried Frailty Phenotype: 0 items: robust; 1 or 2 items: pre-frail; 3 or more items: frail.

*Table S3. Improvement in the number of criteria according to Fried Frailty Criteria and in the FTS-5 score*

|  | *P^a^* | Mean improvement | LL^b^ | UL^c^ |
| --- | --- | --- | --- | --- |
| **Fried Frailty Criteria** | | | | |
| M0-M3 | .09 | 0.34 | -0.05 | 0.74 |
| M0-M6 | .06 | 0.42 | -0.02 | 0.87 |
| **FTS-5** | | | | |
| M0-M3 | .005 | 2.85 | 0.92 | 4.77 |
| M0-M6 | .04 | 2.10 | 0.07 | 4.14 |

a: p-value. b:lower-limit. c: upper-limit.

*Table S4. Secondary outcomes: (Healthcare Resources and Quality of Life):*

| Healthcare Resources Questions | *P^a^* | Beta | LL^c^ | UL^d^ |
| --- | --- | --- | --- | --- |
| Have you experienced any falls in the last 6 months? | .025 | -0.29 | -0.53 | -0.04 |
| How many times have you fallen? | .75 | -0.42 | -2.95 | 2.12 |
| How many times have you visited the emergency room in the last 6 months? | .71 | 0.11 | -0.47 | 0.68 |
| Did you spend any nights in the emergency observation unit? | .25 | -0.20 | -0.51 | 0.12 |
| Have you been hospitalized in the last 6 months? | .30 | -0.11 | -0.33 | 0.10 |
| How many days were you hospitalized? | .77 | 2 | -10.52 | 14.52 |
| How many times have you visited your family doctor in the last 6 months? | .34 | -1.71 | -5.21 | 1.78 |
| How many times have you visited a nurse in the last 6 months? | .30 | -0.92 | -2.64 | 0.80 |
| How many times have you visited a specialist doctor in the last 6 months? | .33 | -0.63 | -1.89 | 0.64 |

| EURO-QoL | *P^a^* | OR^b^ | LL^c^ | UL^d^ |
| --- | --- | --- | --- | --- |
| **M0-M3** | .98 | 0.99 | 0.93 | 1.08 |
| **M0-M6** | .88 | 1.01 | 0.93 | 1.09 |

a: p-value. b: odds-ratio. c:lower-limit. d: upper-limit.
