# Supplementary material for: Effects of Monitoring Frailty Through a Mobile/Web-Based Application and a Sensor Kit to Prevent Functional Decline in Frail and Prefrail Older Adults: FACET (Frailty Care and Well Function) Pilot Randomized Controlled Trial
Source: J Med Internet Res. 2024 Oct 22;26:e58312. doi: 10.2196/58312 (PMC11538877; doi:10.2196/58312)
Supplement: Multimedia Appendix 2 [file jmir_v26i1e58312_app2.docx]

# APPENDIX 2: INFORMED CONSENT

## PARTICIPANT INFORMATION SHEET

### STUDY TITLE: FACET Project: Clinical Validation of the System

Sponsor: Abbott

Principal Investigator: Leocadio Rodriguez-Mañas, MD, PhD

Centers: University Hospital of Getafe and University Hospital Complex of Albacete

### INTRODUCTION

The FACET project aims to provide elderly population and clinical professionals with tools for better management and treatment of FRAILTY. These tools consist of the following:

1. A management, monitoring, and treatment platform for frailty with web access for physicians and mobile device access for the elderly.

• Physicians can:

• Access clinical and demographic information of participants.

• Prescribe and modify treatments based on physical exercise, nutri-tional recommendations, pharmacological recommendations, and questionnaires and tests to be performed at home.

• Monitor the progress of participants and receive alerts if there is a deterioration.

• Participants can:

• Contact the physician through messages via the platform. These messages will be asynchronous, meaning they will not be answered immediately.

• Review prescribed treatments.

• Receive reminders about activities they need to perform as part of their treatment and/or monitoring.

2. A series of sensors installed in the home to record the evolution of the following variables relevant from the perspective of frailty:

• Frailty status.

• Gait speed.

• Chair stands.

• Nutritional status.

• Treatment adherence.

### VOLUNTARY PARTICIPATION

You should know that your participation in this study is voluntary, and you may decide not to participate or change your decision and withdraw consent at any time without affecting your relationship with your doctor or your treatment.

### GENERAL STUDY DESCRIPTION

The study being proposed is the validation of a technological tool that aids in the prevention, treatment, and monitoring of frail and pre-frail patients. The study will take place in the participants' homes and will begin on January 1, 2018, and end on December 31, 2018. Each participant will remain in the study for 6 months.

After signing the informed consent form, participants will be randomized to either the control group or the intervention group:

Control Group: Participants will receive the usual intervention provided in the outpatient geriatric clinic.

Intervention Group: Participants will receive the usual intervention provided in the outpatient geriatric consultation, in addition to having the technological tool described above.

Data from patients in both groups will be collected at 3 time points: in the first month of participation in the study, at 3 months, and at 6 months. In the intervention group, follow-up data will also be collected at home.

The study consists of 8 phases:

1. Selection: Initially, a medical visit will be conducted to ensure that potential participants meet the inclusion and exclusion criteria and agree with the provided information; if so, informed consent will be obtained.

2. Randomization: After signing the informed consent form, participants will be randomly assigned to one of the two groups (control or intervention).

3. Training: If assigned to the intervention group, participants will receive training conducted by the study's technical team to provide information on the use of the platform, sensors, and mobile application.

4. Initial Visit: All study participants, regardless of their assigned group, will undergo the same assessments (Linda Fried criteria, MNA, frailty trait scale, Barthel, frequently asked questions, MMSE, clock test, semantic and phonological fluency, Yasavage, EUROQL-5D, SPPB, gait speed, Up and Go test, Chair Stand Test, healthcare resource utilization). In addition, urine and blood samples (biochemistry and complete blood count) will be collected. This visit will last between 2 and 3 hours.

5. Follow-up: Follow-up data will be collected from participants assigned to the intervention group through questionnaires and different tests to be performed at home using sensors and the mobile application. The responsible physician will analyze the received information and adjust the treatment based on the individual needs of each participant.

6. Follow-up Visit: This visit will take place in the third month of participation and will be conducted for both control and intervention group participants. The visit will be similar to the initial visit and will last between 1.5 and 2 hours.

7. Continuous Monitoring: Follow-up data will continue to be collected from patients assigned to the intervention group. The responsible physician will analyze the received information and adjust the treatment based on the individual needs of each participant.

8. Final Visit: In the 6th month of participation, when the participant exits the study, a new evaluation will be administered. Control group participants will receive an evaluation very similar to the initial one; a urine sample and a blood sample (biochemistry and complete blood count) will be collected. Participants assigned to the intervention group will also need to answer additional questionnaires related to the technology used during the study (SUS, adapted TAM, and satisfaction interview). This final visit will last between 2 and 3 hours.

### POTENTIAL RISKS

The platform, mobile application, and sensors pose minimal risks to the participant, as they have passed all safety checks.

### URINE SAMPLE MANAGEMENT

Samples will be analyzed at the University of Aberystwyth laboratory (Wales) and subsequently destroyed. The analyses performed may reveal information unrelated to the present study; This information will be kept confidential. Since this information is only useful from a research perspective, the test results will not be communicated to you.

This study is conducted in compliance with current legislation regarding the use of biological samples for research purposes (Law 14/2007 on Biomedical Research).

BLOOD SAMPLE HANDLING

Blood samples for analysis (biochemistry and complete blood count) will be collected at visits 1 and 3. These samples will be sent to an external laboratory.

### CONFIDENTIALITY

The processing, communication, and transfer of personal data of all participating subjects will comply with the provisions of Organic Law 15/1999 on the Protection of Personal Data. According to the mentioned legislation, you may exercise your rights of access, modification, opposition, and cancellation of data by contacting your study doctor.

The data collected for the study will be identified by a code, and only the study researchers will be able to relate such data to you. Therefore, your identity will not be disclosed to anyone except under exceptional circumstances such as medical emergencies or legal requirements.

Access to your personal information will be restricted to the study researchers and/or collaborators, the Clinical Research Ethics Committee, and authorized personnel designated by the sponsor, when necessary, to verify the data and procedures of the study, but always maintaining confidentiality in accordance with current legislation.

### ECONOMIC COMPENSATION

This project does not entail any financial benefit for you or the researchers. The study sponsor is responsible for managing its funding.

Participation in the study will not incur any additional expenses for you.

### OTHER RELEVANT INFORMATION

If you decide to withdraw your consent to participate in this study, no new data will be added to the database, and you may request the destruction of all previously retained identifiable documentation to prevent further analysis.

You should also be aware that you may be excluded from the study if the sponsor or study researchers deem it appropriate, either for safety reasons or because they consider that you are not complying with the established procedures. In any case, you will receive an adequate explanation of the reason for your withdrawal from the study.

If you need more information about this study, you can contact the principal investigator of the FACET project, Dr. Leocadio Rodríguez Mañas, Head of the Geriatrics Department at Getafe University Hospital.​
