# Supplementary material for: Effects of Monitoring Frailty Through a Mobile/Web-Based Application and a Sensor Kit to Prevent Functional Decline in Frail and Prefrail Older Adults: FACET (Frailty Care and Well Function) Pilot Randomized Controlled Trial
Source: J Med Internet Res. 2024 Oct 22;26:e58312. doi: 10.2196/58312 (PMC11538877; doi:10.2196/58312)
Supplement: Multimedia Appendix 3 [file jmir_v26i1e58312_app3.pdf]

CONSORT-EHEALTH Checklist V1.6.2 Report	Manuscript Number	58312
**(based on CONSORT-EHEALTH V1.6), available at [http://tinyurl.com/consort-ehealth-v1-6].**		
**Date completed**		
3/26/2024 12:35:01		
**by**		
Rodrigo Pérez-Rodriguez		
TITLE		
**1a-i) Identify the mode of delivery in the title**		
**1a-ii) Non-web-based components or important co-interventions in title**		
"Effect of monitoring frailty through a mobile application and a sensor kit to prevent functional decline in frail and prefrail older people: the FACET pilot randomized control trial."		
**1a-iii) Primary condition or target group in the title**		
ABSTRACT		
**1b-i) Key features/functionalities/components of the intervention and comparator in the METHODS section of the ABSTRACT**		
**1b-ii) Level of human involvement in the METHODS section of the ABSTRACT**		
"Participants were randomized into a control group, receiving standard treatment, and the intervention group, receiving standard treatment along with the home monitoring system (FACET). The system monitored functional test at home (gait speed, chair stand test, frailty status and weight). "		
**1b-iii) Open vs. closed, web-based (self-assessment) vs. face-to-face assessments in the METHODS section of the ABSTRACT**		
**1b-iv) RESULTS section in abstract must contain use data**		
**1b-v) CONCLUSIONS/DISCUSSION in abstract for negative trials**		
INTRODUCTION		
**2a-i) Problem and the type of system/solution**		
**2a-ii) Scientific background, rationale: What is known about the (type of) system**		
In recent decades, the rise in life expectancy has resulted in a larger elderly population, leading to an increase in frail individuals. Consequently, there's a growing interest in preventing and treating frailty due to its challenges for healthcare systems. Current medical practices for assessing and treating frailty rely on periodic visits to healthcare facilities, leaving gaps in monitoring changes in condition, treatment adherence, and response. The FACET technological ecosystem aims to enhance frail patient management by automatically gathering predictive information for adverse events at home, including speed, lower limb power, and involuntary weight loss. This data, along with nutritional assessments and other functional status questionnaires, is uploaded to the platform and provided to geriatric teams, improving effectiveness in frailty management.		
METHODS		
**3a) CONSORT: Description of trial design (such as parallel, factorial) including allocation ratio**		
**3b) CONSORT: Important changes to methods after trial commencement (such as eligibility criteria), with reasons**		
"This research work aims to evaluate the impact of the FACET technological ecosystem when supporting a comprehensive geriatric intervention and follow-up. To do so, a randomized pilot study has been designed to assess whether the information provided by the remote sensors help early detection of functional changes promoting adjusted multimodal intervention in prefrail and frail older persons compared to usual care during a 6-months period."		
**3b-i) Bug fixes, Downtimes, Content Changes**		
**4a) CONSORT: Eligibility criteria for participants**		
"This is a multicentre, randomized, simple blind intervention study, with a duration of 11 months (5 months for recruitment, and 6 for intervention)."<br>"For the participants allocation into either the control or the intervention group, a stratified randomization by age (70-85, >85), sex (male, female), diagnosis (frail and prefrail among Fried Frailty Phenotype Criteria) and educational level (higher education, illiterate, others) was carried out to ensure the 2 research arms are properly balanced."		
**4a-i) Computer / Internet literacy**		
**4a-ii) Open vs. closed, web-based vs. face-to-face assessments:**		
"Finally, to determine if a participant can or cannot understand and use the FACET system, the following procedure will be carried out:<br>1.Provide indications on how to react to a reminder to perform an action through a mobile device of identical conditions to the one that will be used during the experimentation.<br>2.Provide indications on how to fill out a test with the mobile device.<br>3.Generation of a reminder to fill out a test different from the previously shown.<br>If the potential participant reacts to the alarm and completes the test by him/herself, he/she is considered a suitable candidate."		
**4a-iii) Information giving during recruitment**		
Participants were recruited from various settings, including geriatric outpatient clinics, the Acute Care Unit, and Primary Care outpatient clinics, through in-person and telephone approaches. Initially, a pre-screening interview was conducted in the outpatient geriatric facility to identify potentially eligible patients.<br><br>Different researchers were responsible for intervention delivery and data collection during the follow-up. Information collected by the FACET technological ecosystem was reviewed weekly by a non-blinded geriatrician, who then designed and implemented necessary interventions for the intervention group. Data on outcomes were collected through face-to-face interviews every 3 months by another geriatrician, who remained blinded to the participant's randomly allocated branch.		
**4b) CONSORT: Settings and locations where the data were collected**		
The study methodology remained unchanged once initiated.		
**4b-i) Report if outcomes were (self-)assessed through online questionnaires**		
**4b-ii) Report how institutional affiliations are displayed**		
"Data about the outcomes were collected through face-to-face interviews by another geriatrician, who was blinded regarding the branch where the participant had been randomly allocated every 3 months"		
**5) CONSORT: Describe the interventions for each group with sufficient details to allow replication, including how and when they were actually administered**		
**5-i) Mention names, credential, affiliations of the developers, sponsors, and owners**		
**5-ii) Describe the history/development process**		
**5-iii) Revisions and updating**		
**5-iv) Quality assurance methods**		
**5-v) Ensure replicability by publishing the source code, and/or providing screenshots/screen-capture video, and/or providing flowcharts of the algorithms used**		

**5-vi) Digital preservation**		
**5-vii) Access**		
**5-viii) Mode of delivery, features/functionalities/components of the intervention and comparator, and the theoretical framework**		
"The participants in the intervention group accessed to the mobile application through a tablet with 4G connection, provided by the study."		
**5-ix) Describe use parameters**		
The FACET technology consists of two main components:<br><br>Home monitoring subsystem for patients: This includes a mobile application for older individuals, allowing continuous frailty monitoring through a home monitoring kit. The kit records data such as gait speed, lower limb power, and involuntary weight loss, which are processed to trigger alerts for potential deterioration. Patients receive a customized therapeutic plan, including medical treatment, physical activity programs, and nutritional recommendations. They can track their progress, communicate with geriatricians, receive health alarms, and get reminders for home tests.<br><br>Monitoring system: This system serves as a guide for older individuals, a data concentrator, and a data input point for completing questionnaires to enrich clinical information. It includes sensors for measuring variables with predictive value for adverse events, such as gait speed and lower limb power. Collected data are processed to trigger potential deterioration alarms.<br><br>Web interfaces for professionals: This component provides healthcare professionals with infrastructure for accessing and storing clinical information, facilitating messaging with patients, displaying alarms based on monitoring results, tracking patient progress, and managing treatments.		
**5-x) Clarify the level of human involvement**		
**5-xi) Report any prompts/reminders used**		
**5-xii) Describe any co-interventions (incl. training/support)**		
"In the intervention group, the participants were periodically and remotely supervised by their non-blinded geriatrician, who scheduled the questionnaires and tests that the participants must carry out at home to monitor the evolution. Frail Scale, Chair Stand Test, Gait Speed (2.4 meters), and weight were performed once weekly; Barthel Index and FAQ once every 2 weeks and MNA-SF once monthly. These sets of information were captured by the system and stored in the project-dedicated server. The system provides alerts to the clinician (non-blind geriatrician) when pre-established changes were detected. Non-blind geriatrician checked the platform daily to saw if any alarms had been generated. So, in case that an impairment is detected, the geriatrician would call by phone to check the patient's health status and assess them. The non-blind geriatrician, after this phone call could provide an appointment to attend the patient face-to face if needed. When clinically indicated, he/she made changes in the treatment<br>Moreover, the participants and the non-blind researcher could contact by a basic asynchronous communication module along the study."		
**6a) CONSORT: Completely defined pre-specified primary and secondary outcome measures, including how and when they were assessed**		
" The inclusion criteria:<br>•Age ≥ 70 years old.<br>•Living at home.<br>•Having a caregiver or supervision at home.<br>•Barthel index ≥ 90.<br>•Having, at least, 4 comorbidities<br>•Prefrail older adults: Meet 1 or 2 Fried Frailty Phenotype Criteria.<br>•Frail older adults: Meet 3-, 4 or 5 Fried Frailty Phenotype Criteria.<br><br><br>The exclusion criteria were:<br>•Inadequate home infrastructure to host the required technology.<br>•Inability to understand how to use the FACET system.<br>•Illness that impedes carrying out the prescribed therapy and/or the follow-up:<br>oAcute myocardial infarction in the last 3 months.<br>oUnstable cardiovascular disease.<br>oTerminal disease (<12 months of life expectancy).<br>oOther pathologies involving clinical instability.<br>•Alcohol/drugs abuse.<br>•Living with a participant in the trial.<br>•Participation in other interventional clinical studies at the same time."		
**6a-i) Online questionnaires: describe if they were validated for online use and apply CHERRIES items to describe how the questionnaires were designed/deployed**		
**6a-ii) Describe whether and how "use" (including intensity of use/dosage) was defined/measured/monitored**		
**6a-iii) Describe whether, how, and when qualitative feedback from participants was obtained**		
**6b) CONSORT: Any changes to trial outcomes after the trial commenced, with reasons**		
"Recruitment, assessment, follow-up, and treatment was carried out in parallel in 2 institutions: Getafe University Hospital and Albacete University Hospital."		
**7a) CONSORT: How sample size was determined**		
**7a-i) Describe whether and how expected attrition was taken into account when calculating the sample size**		
**7b) CONSORT: When applicable, explanation of any interim analyses and stopping guidelines**		
"Data were collected from participants during face-to-face visits in outpatient clinics, at baseline, 3 and 6 months, except cognitive test that were assessed at baseline and at 6 months. Data collected were the following:<br><br><br>1. Demographic data.<br>2. Comorbidities<br>3. Medication.<br>4. Barthel index(9)<br>5. Frail Phenotype Criteria(10)<br>6. Frailty Trait Scale 5 (31)<br>7. Gait speed in 6 meters(11)<br>8. Mini Mental Status Examination (MMSE)(12).<br>9. EuroQL-5D-5L(13)<br>10. Use of health resources in the last 6 months. "		
**8a) CONSORT: Method used to generate the random allocation sequence**		
No changes were made to the primary or secondary objectives once the study had commenced.		
**8b) CONSORT: Type of randomisation; details of any restriction (such as blocking and block size)**		
No interim analyses were conducted.		
**9) CONSORT: Mechanism used to implement the random allocation sequence (such as sequentially numbered containers), describing any steps taken to conceal the sequence until interventions were assigned**		
 The randomization was performed using the MINIM tool.		
**10) CONSORT: Who generated the random allocation sequence, who enrolled participants, and who assigned participants to interventions**		

"For the participants allocation into either the control or the intervention group, a stratified randomization by age (70-85, >85), sex (male, female), diagnosis (frail and prefrail among Fried Frailty Phenotype Criteria) and educational level (higher education, illiterate, others) was carried out to ensure the 2 research arms are properly balanced.<br>A recruitment target of 90 participants was established. 44 participants were allocated in control group and 46 in intervention group."		
**11a) CONSORT: Blinding - If done, who was blinded after assignment to interventions (for example, participants, care providers, those assessing outcomes) and how**		
**11a-i) Specify who was blinded, and who wasn't**		
**11a-ii) Discuss e.g., whether participants knew which intervention was the "intervention of interest" and which one was the "comparator"**		
"-Non-blind geriatricians: check and verify participant's eligibility based on the inclusion and exclusion criteria. Explain the study details and obtain informed consent before randomization. Monitor progress through the FACET system and adjust the treatment as needed along the study.<br>-Blind geriatricians: they participated in the pre-randomization tasks of the study. They did not received information about the arm to which the patient was randomly allocated. They also made the participant's evaluations at the baseline visit, month 3 and month 6. These researchers did not have access to the data from FACET monitoring system.<br><br>-Biomedical engineer: training sessions with the participants on the use of the FACET monitoring system. Installation of technology at home. Resolution of technical problems during the development of the study."		
**11b) CONSORT: If relevant, description of the similarity of interventions**		
No sequence was initially creted but participans were allocated using the MINIM tool configured according to the study needs		
**12a) CONSORT: Statistical methods used to compare groups for primary and secondary outcomes**		
https://classic.clinicaltrials.gov/ct2/show/NCT03707145		
**12a-i) Imputation techniques to deal with attrition / missing values**		
**12b) CONSORT: Methods for additional analyses, such as subgroup analyses and adjusted analyses**		
"Control group (n=44) received usual geriatric care through the classical ways of providing it (comprehensive geriatric assessment, adjustment of polypharmacy, physical, cognitive and nutritional prescription done face-to-face in classical patients visits). Participants in the intervention group (n=46) received the usual health care by a geriatric team, but supported by the information provided by the FACET monitoring system. "		
RESULTS		
**13a) CONSORT: For each group, the numbers of participants who were randomly assigned, received intended treatment, and were analysed for the primary outcome**		
Provide a more extensive description of randomization.		
**13b) CONSORT: For each group, losses and exclusions after randomisation, together with reasons**		
No additional analyses were performed.		
**13b-i) Attrition diagram**		
**14a) CONSORT: Dates defining the periods of recruitment and follow-up**		
"Recruitment ceased when 90 participants were involved. Randomization resulted in the allocation of 44 participants into the control group, and 46 into the intervention group. Out of the whole sample of 90 subjects. "		
**14a-i) Indicate if critical "secular events" fell into the study period**		
**14b) CONSORT: Why the trial ended or was stopped (early)**		
We have included a CONSORT flow diagram in our manuscript to illustrate the flow of participants through each stage of the trial, including enrollment, allocation, follow-up, and analysis.<br>In the third month of follow-up, 3 participants from de control group refused to continue in the study and 7 in the intervention group. In the follow-up at month 6, 2 participants from the intervention group dropped out (1 did not want to continue and 1 critically ill). So, from the original sample, 41 participants from the control group and 37 from the intervention group completed the full study.		
**15) CONSORT: A table showing baseline demographic and clinical characteristics for each group**		
"Duration of 11 months (5 months for recruitment, and 6 for intervention). The study was conducted from August 2018 to June 2019"		
**15-i) Report demographics associated with digital divide issues**		
**16a) CONSORT: For each group, number of participants (denominator) included in each analysis and whether the analysis was by original assigned groups**		
**16-i) Report multiple "denominators" and provide definitions**		
**16-ii) Primary analysis should be intent-to-treat**		
This information can be found in article: "Pérez-rodríguez R, Villalba-mora E, Valdés-aragonés M, Ferre X, Moral-martos C, Mas-romero M, et al. Usability, user experience, and acceptance evaluation of capacity: A technological ecosystem for remote follow-up of frailty. Sensors. 2021;21(19). "		
**17a) CONSORT: For each primary and secondary outcome, results for each group, and the estimated effect size and its precision (such as 95% confidence interval)**		
The trial was not stopped prematurely.		
**17a-i) Presentation of process outcomes such as metrics of use and intensity of use**		
**17b) CONSORT: For binary outcomes, presentation of both absolute and relative effect sizes is recommended**		
"Table 1. Overall Group Description<br>A table is presented in the manuscript with the demographic characteristics of the participants.<br>"The baseline characteristics of the participants are summarized in table 1. Both groups exhibited similar baseline traits. The mean age of our study population was 82.33 years ±5.91 years. The majority were women (72.22%), had limited education (either no education or only primary studies), and did not use technology daily.<br><br>Participants in both groups showed independency in basic activities of daily living, with a mean Barthel Index (94.11 ± 3.72). Participants met a mean of 2.73 ± 0.86 criteria and 50% where categorized as frail and 50% as prefrail. The mean score of FTS-5 was 22.89 ± 6.14.<br>Regarding functional status, gait speed, SPPB, and test up and go indicated mild levels of physical impairment, whereas the chair stand test (CST) results showed a moderate level of physical impairment. Mean cognitive status values indicated very mild cognitive impairment, and the sample exhibited a low mood level<br>No significant differences were observed between the two groups, confirming the adequacy of the randomization process."		
**18) CONSORT: Results of any other analyses performed, including subgroup analyses and adjusted analyses, distinguishing pre-specified from exploratory**		

1. Frailty worsening:
A) Changes in FTS-5 score and worsening of 1 criterion in the Fried Frailty Phenotype Criteria: when we compared the intervention and control group along the time through changes in the FTS-5, we observed a 77% reduction in the risk of deterioration at the limits of statistical significance (OR=0.23, 95% IC= 0.05-1.09; p=0.06) at 3 months in the intervention group that reached the statistical significance at 6 months of follow-up, with a 74% reduction in the risk of deterioration (OR=0.26, 95% IC = 0.07 – 0.98; p-value = 0.04) (Table 2).

When analysing data based on a 1 point worsening according to the Fried Frailty Phenotype Criteria, the results showed that the intervention group resulted in a 92% lower likelihood of worsening compared to the control group at 6 month follow-up (OR=0.08, 95% CI=0.01-0.67; p-value=0.02) (Table 2). As there were not events at three months in any of the two groups it was not possible to assess the effect.

Frailty transitions were not significantly altered at 3 or 6 months on either FTS-5 or Fried Frailty Phenotype Criteria scales. However, in terms of frailty improvement:

For FTS-5, there was a higher likelihood of improvement in the intervention group compared to the control group at both 3 and 6 months.
According to Fried Frailty Phenotype Criteria, improvement was observed at 3 months in the intervention group compared to the control group, but not at 6 months.
Regarding transitions from frail to non-frail or from pre-frail to robust, there were no significant results observed by FTS-5 at either time point. However, analyzing transitions by Fried Frailty Phenotype Criteria showed a marginal higher likelihood of improvement in frailty status in the intervention group compared to the control group at 3 months, but not at 6 months.

In terms of secondary outcomes, no significant changes were observed in visits to the Emergency Room, hospitalizations, visits to Primary Care physicians and nurses, number of falls, or Quality of Life. More detailed information can be found in Appendix 2.

18-i) Subgroup analysis of comparing only users

19) CONSORT: All important harms or unintended effects in each group

The effect of the intervention is analyzed in a binary manner (based on the study objectives) in the main document and in a continuous manner in the annexes.

19-i) Include privacy breaches, technical problems

19-ii) Include qualitative feedback from participants or observations from staff/researchers

DISCUSSION

20) CONSORT: Trial limitations, addressing sources of potential bias, imprecision, multiplicity of analyses

20-i) Typical limitations in ehealth trials

21) CONSORT: Generalisability (external validity, applicability) of the trial findings

21-i) Generalizability to other populations

21-ii) Discuss if there were elements in the RCT that would be different in a routine application setting

22) CONSORT: Interpretation consistent with results, balancing benefits and harms, and considering other relevant evidence

22-i) Restate study questions and summarize the answers suggested by the data, starting with primary outcomes and process outcomes (use)

22-ii) Highlight unanswered new questions, suggest future research

"Participants allocated to the intervention group showed a 73% reduction in the risk of deterioration by FTS-5 score (p-value = 0.04) and 92% lower likelihood of worsening by 1 point according to Fried Frailty Phenotype Criteria compared to the control group (p-value = 0.02) at 6-months of follow-up. Frailty status, when assessed through FTS-5, improved in the intervention group at 3 (p-value = 0.004) and 6 months (p-value = 0.047), while when the Frailty Phenotype Criteria were used, benefits were shown at 3 months of follow-up (p-value = 0.03), but not at 6 months. "

Other information

23) CONSORT: Registration number and name of trial registry

No additional analyses were performed.

24) CONSORT: Where the full trial protocol can be accessed, if available

Twenty-six adverse events unrelated to the use of the FACET system were reported. These included hospital admissions for influenza A virus infection, obstructive chronic pulmonary disease exacerbation, rectal bleeding, and moderate haemoptysis. Most adverse events were emergency department visits for various symptoms or diagnoses, such as lower respiratory tract infections, heart failure, urinary tract infections, tendinitis, arthritis, allergic reactions, gait disturbances, falls with cranioencephalic trauma, hypoglycaemia, hematuria, and knee pain. Additionally, two participants underwent outpatient cataract surgery.

25) CONSORT: Sources of funding and other support (such as supply of drugs), role of funders

The study was registered in clinical trial gov (NCT03707145). Frailty care and well-function in community dwelling older adults (FACET)

X26-i) Comment on ethics committee approval

x26-ii) Outline informed consent procedures

X26-iii) Safety and security procedures

X27-i) State the relation of the study team towards the system being evaluated
